# Validating women’s reports of antenatal and postnatal care received in Bangladesh, Cambodia and Kenya

**DOI:** 10.1136/bmjgh-2019-002133

**Published:** 2020-04-19

**Authors:** Katharine J McCarthy, Ann K Blanc, Charlotte Warren, Ashish Bajracharya, Benjamin Bellows

**Affiliations:** 1 Department of Epidemiology and Biostatistics, CUNY Graduate School of Public Health & Health Policy, New York, New York, USA; 2 Population Council, New York, New York, USA; 3 Population Council, Washington, District of Columbia, USA; 4 Population Council, Phnom Penh, Cambodia

**Keywords:** health policy, maternal health, paediatrics, health systems evaluation

## Abstract

**Background:**

Global indicators for monitoring progress in maternal and newborn health have tended to rely on contact coverage indicators rather than the content of services received. As part of the effort to improve measurement of progress in maternal and newborn health, this study examines how accurately women can report on information and health interventions received during an antenatal or postnatal health consultation at health facilities in Bangladesh, Cambodia and Kenya.

**Methods:**

We conducted secondary analysis of matched observation and client interview data to compare women’s reports of care received at exit interview with observation by a trained third-party observer. We assessed indicator accuracy by calculating sensitivity, specificity, area under the receiver operating characteristic curve (AUC) and inflation factor (IF). Indicators considered to have both high individual accuracy (an AUC value of 0.70 or greater) and low population-level bias (0.75<IF<1.25) were considered to have acceptable validity. In addition, we considered the number of countries where both validation criteria were met.

**Results:**

For indicators of antenatal care, we found 16 of 18 indicators in Bangladesh, 3 of 6 in Cambodia and 3 of 8 in Kenya met both validation criteria. For postnatal care, we found evidence of acceptable validity for 6 of 8 indicators in Bangladesh, 5 of 14 in Cambodia and 3 of 16 in Kenya. In general, we documented higher validity for indicators related to concrete, observable actions, as opposed to information or advice given. Women were more likely to recall care received for themselves, rather than for their newborn.

**Conclusions:**

Women reported accurately on multiple aspects of antenatal and postnatal care. While we describe broad patterns in the types of indicators likely to be recalled with accuracy, differences by setting warrant further investigation. Findings inform efforts to better monitor the coverage and quality of maternal and newborn health interventions.

**Key questions**

**What is already known?**

- Self-reported data obtained in population-based household surveys are frequently used to determine global and national coverage for maternal and newborn health interventions (ie, the proportion of individuals in need of an intervention who receive it).
- Prior validation studies have suggested that, with few exceptions, women are not able to report accurately on care received in the intrapartum or immediate postnatal period (up to 1 hour following birth).
- Few studies have examined how accurately women can report on the content of care received during an antenatal or postnatal health consultation for themselves or their newborn.

**What are the new findings?**

- Validation analyses of women’s immediate recall of facility-based care across three countries suggest that women can accurately report on several interventions in the antenatal and postnatal periods; however, there were differences by setting.
- Women were more likely to report accurately on concrete, observable interventions as opposed to information or advice given.

**What do the new findings imply?**

- In the context of calls for enhanced measurement of the components that lead to effective coverage, findings suggest that careful consideration of the type of information women are asked to recall is needed.
- As new indicators are proposed, they should be subject to validity tests using variations in wording, recall period and setting.

## Introduction

Service contact indicators such as attending antenatal care (ANC) within the first 14 weeks of gestation, delivering at a health facility and receipt of postnatal care (PNC) in the first 2 days of birth have been widely used to track progress towards national and international health goals.^1^ In many low-income and middle-income countries (LMICs), household surveys are the best or only available data on the coverage of maternal and newborn services.^2^ Household survey programmes such as the Demographic and Health Surveys (DHS) and Multiple Indicator Cluster Surveys (MICS) collect information from women via a series of questions in a face-to-face interview. Yet there are measurement challenges associated with these indicators.

A growing body of literature highlights the wide discrepancies between indicators of service contact and those that measure receipt of high-quality services and associated benefits in maternal and newborn care.^3–7^ For example, an analysis of DHS data from 41 countries found that, among women reporting four or more ANC visits for a birth in the preceding 2 years, a substantial percentage of women did not receive the recommended services, with a gap between expected and actual coverage ranging from 18% to 86% across countries.^6^ To highlight such gaps, a coverage cascade framework that identifies key losses in service quality has been proposed to measure effective coverage.^3^ Similarly, updated WHO guidelines for maternal and newborn care emphasise the quality and provision of appropriate and timely interventions (quality-adjusted coverage) in addition to service contact indicators.^8 9^ Further, several global strategies such as the Global Strategy for Women’s, Children’s and Adolescents’ Health,^10^ Every Newborn Action Plan,^11^ and Ending Preventable Maternal Mortality, in addition to the Sustainable Development Goal agenda,^12^ have set renewed targets to improve maternal and newborn health by 2030. These initiatives also include indicators (either in use or under development) that directly relate to the quantity and quality of care of women and newborns.^1^

Despite this, few questions in surveys such as MICS and DHS reflect women’s receipt of specific health interventions. Most of the questions relate to antenatal and intrapartum interventions. ANC service coverage interventions currently measured in DHS, for example, include an ANC visit in the first trimester, measurement of blood pressure, tetanus toxoid vaccination, urine testing, counselling about danger signs, HIV counselling and testing, iron-folate supplementation, and malaria prevention. In contrast, despite accounting for more maternal deaths than any other phase of pregnancy, no PNC interventions for the mother and few for the newborn are currently tracked in the DHS or MICS. More recently, there have been efforts to change this omission. For example, an optional module on pregnancy and PNC was recently added to the DHS. Further, several aspects of newborn PNC have been recently added to the core DHS-7 questionnaires. These include whether within the first 2 days of birth the provider examined the cord, measured the infant’s temperature, counselled the mother on danger signs for newborns and counselled/observed the mother breast feeding.

While the expansion in routinely tracked maternal and newborn indicators is encouraging, data availability must be weighed against the accuracy of what is measured. A growing, although limited, body of research has sought to assess the validity of women’s recall of maternal and newborn health interventions by comparing women’s reports with a ‘gold standard’ measure. Recent research has used observations by a trained observer as the reference standard.^13–18^ Taken together, these studies suggest that women are not likely to accurately recall interventions received during the intrapartum or immediate postnatal period (within an hour of birth).^13–15 17^ A study of similar design that assessed women’s immediate recall following a return PNC health visit in Kenya and eSwatini suggests that the accuracy of women’s reports is greater for select postnatal indicators relative to intrapartum and immediate postnatal indicators, although there were some differences by setting.^16^ A study conducted in rural China compared women’s recall of ANC and PNC with medical records rather than observer report.^19^ To the best of our knowledge, this is also the only study to date to have also examined the validity of reporting of interventions received as part of routine ANC, which were generally found to be recalled with poor specificity.^19^

Given the renewed focus on measurement of quality-adjusted coverage, as well as the limited number of studies that have sought to assess self-reported antenatal or postnatal care interventions, additional validation work is warranted. The present study aims to extend research findings to date by assessing the validity of a set of antenatal and postnatal service coverage indicators that reflect a range of recommended interventions and counselling procedures across three different settings.

## Methods

We compared women’s reports of antenatal or postnatal care received against observations by a trained third-party observer using a structured checklist in health facilities located in Bangladesh, Cambodia and Kenya. Women’s reports of care received were collected via exit interview prior to them leaving the health facility following a health visit for themselves or their newborn. Data were originally collected as part of an evaluation of a voucher and accreditation intervention led by the Population Council in Bangladesh, Cambodia and Kenya, with the National Institute of Public Health in Cambodia and Research Training and Management (RTM) International in Bangladesh as partners. The primary objective of the evaluation was to assess the influence of the voucher programme (henceforth ‘Voucher programme’) on maternal and newborn health service utilisation, including antenatal and postnatal care. A secondary objective was to determine whether the voucher programme improved service quality by verifying service delivery through reimbursements to providers. Country-specific study protocols that detail data collection processes have been previously published.^20–22^ As the study relied on extant data, indicators were not identified a priori. Survey questionnaires were reviewed for indicators which were reported by both the observers and the women for validation purposes. Online supplementary table 1 displays which indicators align with global and national development goals, as well as those which have the potential to be included in such efforts. Patients or the public were not involved in the design, or conduct, or reporting, or dissemination plans of our research.

10.1136/bmjgh-2019-002133.supp1 Supplementary data

### Study settings

Matched exit interviews and observations were conducted in 124 health facilities across three countries: Bangladesh (22 facilities), Cambodia (40 facilities) and Kenya (62 facilities). Two rounds of cross-sectional data were collected in each country: between 2011 and 2013 in Bangladesh, and between 2010 and 2012 in Cambodia and Kenya. In each setting, approximately half of the facilities were accredited to provide maternal and newborn health and family planning services to women holding vouchers for the services (ie, voucher intervention facilities). Voucher facilities were compared with a sample of non-accredited (control) facilities from the same/similar districts. To reduce the potential for selection bias related to facility enrolment, pairwise matching was also used to match facilities on factors hypothesised to influence provider behaviour a priori, including profile of clientele, location and fees charged, type of practice, and skills mix.^21 22^ In total, 3169 women were interviewed and observed for ANC (n=1036 in Bangladesh, 957 in Cambodia and 1176 in Kenya) and 2462 for PNC (n=208 in Bangladesh, 635 in Cambodia and 1619 in Kenya).

In Bangladesh, all health facilities were government upazila health complexes, located in 22 upazilas (subdistricts) from six divisions: Barisal, Chittagong, Dhaka, Khulna, Rajshahi and Sylhet. The majority (77%) of facilities offered comprehensive obstetric care, while about a quarter offered basic obstetric care (23%). More than half of facilities had a skilled provider for caesarean delivery and six had capacity for blood transfusion. Out of the 22 facilities, 17 provided referral to a district hospital or maternal and child welfare centre. Nationally, 29% of deliveries to women took place in a health facility in the 3 years preceding the 2011 DHS, 26% of pregnant women received four or more antenatal visits, and 28% of women received postnatal health check in the first 2 days of birth, while more than nearly-two thirds of mothers received no PNC (61%).^23^

In Cambodia, 40 public/government health facilities were located in 5 provinces (Kampong Thom, Kampot Speu, Prey Veng, Kampot, and Takeo). All but two health facilities were health centres; two were former district hospitals. Most facilities (68%) had a single bed. Half of facilities were located 15 km or less from the nearest referral hospital, while 35% were located at a distance of 10 km or less. At the national level, 54% of women with a birth in the preceding 5 years delivered in a health facility, 60% of pregnant women reported receiving four or more ANC visits, and 70% of women who gave birth in the 5 years before the interview received PNC within the first 2 days of delivery.^24^ Of the mothers, 26% received no PNC. Of the provinces where data collection took place, PNC was notably lower than the national average: reports of no PNC ranged from 17% in Kampot/Kep to 53% in Oddor Mean Chey.^24^

In Kenya, 62 facilities were located in Kisumu, Kiambu, Kitui counties, and the Korogocho and Viwandani informal settlements in Nairobi. Facilities were either public (n=40), private-for-profit (n=10), faith-based (n=9) or non-governmental organisation (n=3). The majority of facilities were hospitals (61%), followed by health centres (31%) and nursing homes, dispensaries or clinics (8%). The national prevalence of facility delivery in Kenya was 43% at the time of the most recent DHS survey that preceded data collection (2008–2009), 47% of pregnant women reported receiving four or more ANC visits, and 42% of women who gave birth in the 5 years preceding the survey received postnatal check-up within 2 days of birth, while 53% received no PNC.^25^

### Data management

In Cambodia and Kenya, unique identification codes for the client exit interview and observation record of received care were matched. In Bangladesh, identification codes were generated by combining information on facility, date, type of service received and end time of observation/start time of interview. If there was ambiguity (a time lag of more than 45 min or more than one feasible match), the case was excluded. The process resulted in 1244 matches out of 2228 cases (56% match) (1036 cases for ANC and 208 for PNC). We performed a sensitivity analysis by restricting the maximum time difference to 30 min and expanding it to 60 min, with minimal impact on sample size (<50 case difference). Given the conservative approach used, we are confident in the accuracy of the matching process.

Data for each cross-sectional year of data collection were pooled for each country. Questions about whether interventions occurred were coded 1 if the response was ‘Yes’ and 0 if ‘No’.

### Sample size

We anticipated indicator prevalence would range between 50% and 80% coverage because assessed indicators were health-promoting rather than harmful practices. We assumed levels of moderate to high sensitivity (60%–70%) and specificity (70%–80%), given recall was immediate. The sample size for anticipated sensitivity and specificity levels was calculated using Buderer’s formula.^26^ We set α=0.05 for both accuracy parameters assuming a normal approximation to a binomial distribution. Based on these specifications, a sample size of 400 women per country is sufficient to estimate 60% sensitivity and 70% specificity with at least 7% precision.

### Validation analysis

We constructed two-by-two tables and calculated sensitivity (the true positive rate) and specificity (true negative rate) for each indicator. A greater number of indicators were assessed in Bangladesh due to differences in questionnaires, which allowed more aspects of care to be matched between observer and client reports. ‘Don’t Know’ responses were excluded in validity estimates but are reported in tables 1 and 2. We estimated the area under the receiver operating characteristic curve (AUC) and corresponding 95% CI following a binomial distribution. The AUC can be interpreted as ‘the average sensitivity across all possible specificities’.^27^ An AUC of 0.5 indicates an uninformative test and an AUC of 1.0 represents perfect accuracy (100% sensitivity and 100% specificity).^27^ An AUC value of 0.7 or higher was used as the cut-off criteria for high individual-level reporting accuracy.^28^

**Table 1:** ANC validation results

Indicator	Study	Client-reported (n)	Don’t know (n)	n	TP	FP	FN	TN	Observed (true) prevalence	Estimated prevalence	Sensitivity (95% CI)	Specificity (95% CI)	AUC (95% CI)	IF	Met both criteria?	No. studies both criteria met
Measure weight	BA	1035	0	1035	657	23	23	332	65.7	65.7	96.6 (95.0 to 97.8)	93.5 (90.4 to 95.8)	0.951 (0.94 to 0.97)	1.00	Y	*
	CA	958	0	949	828	54	40	27	91.5	92.9	95.4 (93.8 to 96.7)	33.3 (23.2 to 44.7)	0.644 (0.59 to 0.70)	1.02	N	
	KE	779	1	776	717	17	18	24	94.7	94.6	97.6 (96.2 to 98.5)	58.5 (42.1 to 73.7)			NA	
Blood pressure check	BA	1035	0	1035	907	19	18	91	89.4	89.5	98.1 (96.9 to 98.8)	82.7 (74.3 to 89.3)	0.904 (0.87 to 0.94)	1.00	Y	*
	CA	960	0	951	920	15	10	6	97.8	98.3	98.9 (98.0 to 99.5)	28.6 (11.3 to 52.2)			NA	
	KE	1158	0	1156	1031	37	54	34	93.9	92.4	95.0 (93.6 to 96.2)	47.9 (35.9 to 60.1)	0.715 (0.66 to 0.77)	0.98	Y	
Examine abdomen	BA	1035	5	1030	703	44	24	259	70.7	72.5	96.7 (95.2 to 97.9)	85.5 (81.0 to 89.2)	0.911 (0.89 to 0.93)	1.03	Y	*
	CA	959	0	950	733	79	49	90	82.3	85.4	93.7 (91.8 to 95.3)	53.6 (45.7 to 61.3)	0.737 (0.70 to 0.78)	1.04	Y	
	KE	1158	0	1156	1086	23	38	9	97.2	95.9	96.6 (95.4 to 97.6)	28.1 (13.7 to 46.7)			NA	
Check anaemia (pallor or refer to HB test)	BA	1035	0	1035	635	53	75	272	68.6	66.5	89.4 (86.9 to 91.6)	83.7 (79.2 to 87.5)	0.866 (0.84 to 0.89)	0.97	Y	*
	CA	957	0	948	375	127	101	345	50.2	52.9	78.8 (74.8 to 82.4)	73.1 (68.8 to 77.0)	0.759 (0.73 to 0.79)	1.05	Y	
	KE	1157	17	1139	568	107	314	150	77.4	59.3	64.4 (61.1 to 67.6)	58.4 (52.1 to 64.5)	0.614 (0.58 to 0.65)	0.77	N	
Check fetal heart rate	BA	1034	2	1032	508	56	58	410	54.8	54.6	89.8 (87.0 to 92.1)	88.0 (84.7 to 90.8)	0.889 (0.87 to 0.91)	1.00	Y	*
	CA	959	0	949	286	57	122	484	43.0	36.2	70.1 (65.4 to 74.5)	89.5 (86.6 to 91.9)	0.798 (0.77 to 0.82)	0.84	Y	
	KE	1158	4	1152	946	105	46	55	86.1	91.2	95.4 (93.9 to 96.6)	34.4 (27.1 to 42.3)	0.649 (0.61 to 0.69)	1.06	N	
Urine screen	BA	1032	0	1032	203	25	94	710	28.8	22.1	68.4 (62.7 to 73.6)	96.6 (95.0 to 97.8)	0.825 (0.80 to 0.85)	0.77	Y	*
	CA	956	0	947	1	31	4	911	0.5	3.4	20.0 (0.5 to 71.6)	96.7 (95.4 to 97.8)			NA	
	KE	1157	0	1153	313	179	100	561	35.8	42.7	75.8 (71.4 to 79.8)	75.8 (72.6 to 78.9)	0.758 (0.73 to 0.78)	1.19	Y	
Inform status of pregnancy	CA	956	0	947	190	300	98	359	30.4	51.7	66.0 (60.2 to 71.4)	54.5 (50.6 to 58.3)	0.602 (0.57 to 0.64)	1.70	N	
	KE	1158	3	1153	790	201	107	55	77.8	85.9	88.1 (85.8 to 90.1)	21.5 (16.6 to 27.0)	0.548 (0.52 to 0.58)	1.10	N	
Give return date	CA	955	0	945	793	58	62	32	90.5	90.1	92.7 (90.8 to 94.4)	35.6 (25.7 to 46.3)	0.642 (0.59 to 0.69)	1.00	N	
	KE	1586	5	1149	991	36	104	18	95.3	89.4	90.5 (88.6 to 92.2)	33.3 (21.1 to 47.5)	0.619 (0.56 to 0.68)	0.94	N	
Provider: nurse or midwife	BA	1036	0	1036	158	15	6	857	15.8	16.7	96.3 (92.2 to 98.6)	98.3 (97.2 to 99.0)	0.973 (0.96 to 0.99)	1.06	Y	*
	KE	1158	0	1155	992	32	67	64	91.7	88.7	93.7 (92.0 to 95.1)	66.7 (56.3 to 76.0)	0.802 (0.75 to 0.85)	0.97	Y	
Provider: doctor/medical officer	BA	1036	0	1036	466	7	17	546	46.6	45.6	96.5 (94.4 to 97.9)	98.7 (97.4 to 99.5)	0.976 (0.97 to 0.99)	0.98	Y	*
	KE	1158	0	1155	38	112	1	1004	3.4	13.0	97.4 (86.5 to 99.9)	90.0 (88.0 to 91.7)	0.937 (0.91 to 0.96)	3.83	N	
Indicators measured in Bangladesh only																
Take pulse	BA	1034	0	1022	281	103	58	580	33.2	37.6	82.9 (78.5 to 86.7)	84.9 (82.0 to 87.5)	0.839 (0.81 to 0.86)	1.13	Y	*
Check temperature	BA	1035	0	1035	236	63	87	649	31.2	28.9	73.1 (67.9 to 77.8)	91.2 (88.8 to 93.1)	0.821 (0.79 to 0.85)	0.93	Y	*
Vaginal examination	BA	1035	0	1031	11	24	13	983	2.3	3.4	45.8 (25.6 to 67.2)	97.6 (96.5 to 98.5)			NA	
Perform/refer for blood test	BA	1034	0	1034	239	14	95	686	32.3	24.5	71.6 (66.4 to 76.3)	98.0 (96.7 to 98.9)	0.848 (0.82 to 0.87)	0.76	Y	*
Perform/refer for ultrasonogram	BA	1033	0	1033	143	16	113	761	24.8	15.4	55.9 (49.5 to 62.0)	97.9 (96.7 to 98.8)	0.769 (0.74 to 0.80)	0.62	N	
Check oedema	BA	1035	0	1024	459	64	70	431	51.7	51.1	86.8 (83.6 to 89.5)	87.1 (83.8 to 89.9)	0.869 (0.85 to 0.89)	0.99	Y	*
Advise on diet/nutrition	BA	1034	0	1034	566	110	67	291	61.2	65.4	89.4 (86.8 to 91.7)	72.6 (67.9 to 76.9)	0.810 (0.78 to 0.83)	1.07	Y	*
Advise on TT injection	BA	1034	0	1034	448	89	70	427	50.1	51.9	86.5 (83.2 to 89.3)	82.8 (79.2 to 85.9)	0.846 (0.82 to 0.87)	1.04	Y	*
Discuss care for breasts/breast feeding	BA	485	0	483	24	22	14	423	7.9	9.5	63.2 (46.0 to 78.2)	95.1 (92.6 to 96.9)			NA	
Inform on possible pregnancy-related complications	BA	1034	0	1034	121	265	38	610	15.4	37.3	76.1 (68.7 to 82.5)	69.7 (66.5 to 72.7)	0.729 (0.70 to 0.76)	2.51	N	
Family welfare visitor attended ANC	BA	1036	0	1036	337	14	11	674	33.6	33.9	96.8 (94.4 to 98.4)	98.0 (96.6 to 98.9)	0.974 (0.96 to 0.98)	1.01	Y	*
Medical assistant attended ANC	BA	1036	0	1036	30	7	11	988	4.0	3.6	73.2 (57.1 to 85.8)	99.3 (98.6 to 99.7)	0.862 (0.79 to 0.93)	0.90	Y	*

Inflation factor (IF) is the ratio of estimated prevalence that would be obtained from a household survey applying the sensitivity and specificity to the prevalence observed in this study to the true (observed) prevalence.

Benchmark for high validity is AUC >0.7 and IF between 0.75 and 1.25.

See online supplementary tables 1 and 2 for indicator construction and differences across studies.

*Number of asterisks refers to number of studies where both criteria were met.

ANC, antenatal care; AUC, area under the receiver operating characteristic curve; BA, Bangladesh; CA, Cambodia; FN, false negative; FP, false positive; FWV, family welfare visitor; HB, haemoglobin; KE, Kenya; N, no; NA, not assessed; TN, true negative; TP, true positive; TT, tetanus toxoid; Y, yes.

**Table 2:** PNC validation results

Indicator	Study	Client-reported (n)	Don’t know (n)	Matched (n)	TP	FP	FN	TN	Observed (true) prevalence	Estimated prevalence	Sensitivity (95% CI)	Specificity (95% CI)	AUC (95% CI)	IF	Met both criteria?	No. studies both criteria met†
Maternal care																
Blood pressure check	BA	209	0	208	49	2	2	155	24.5	24.5	96.1 (86.5 to 99.5)	98.7 (95.5 to 99.8)	0.974 (0.95 to 1.00)	1.00	Y	*
	CA	633	0	632	523	29	18	62	85.6	87.3	96.7 (94.8 to 98.0)	68.1 (57.5 to 77.5)	0.824 (0.78 to 0.87)	1.02	Y	
	KE	1585	4	1577	268	216	104	989	23.6	30.7	72.0 (67.2 to 76.5)	82.1 (79.8 to 84.2)	0.771 (0.75 to 0.80)	1.30	N	
Breast examination	BA	209	0	208	7	4	2	195	4.3	5.3	77.8 (40.0 to 97.2)	98.0 (94.9 to 99.4)			NA	*
	CA	633	0	632	267	75	50	240	50.2	54.1	84.2 (79.7 to 88.1)	76.2 (71.1 to 80.8)	0.802 (0.77 to 0.83)	1.08	Y	
	KE	1585	1	1580	153	202	70	1155	14.1	22.5	68.6 (62.1 to 74.6)	85.1 (83.1 to 87.0)	0.769 (0.74 to 0.80)	1.59	N	
Examine abdomen	BA	209	0	208	6	4	10	188	7.7	4.8	37.5 (15.2 to 64.6)	97.9 (94.8 to 99.4)			NA	*
	CA	633	0	632	395	97	38	102	68.5	77.8	91.2 (88.2 to 93.7)	51.3 (44.1 to 58.4)	0.712 (0.68 to 0.75)	1.14	Y	
	KE	1585	0	1582	201	269	33	1079	14.8	29.7	85.9 (80.8 to 90.1)	80.0 (77.8 to 82.1)	0.830 (0.8 to 0.85)	2.01	N	
Examine vagina	BA	209	0	208	35	10	16	147	24.5	21.6	68.6 (54.1 to 80.9)	93.6 (88.6 to 96.9)	0.811 (0.74 to 0.88)	0.88	Y	*
	CA	633	0	632	234	110	25	263	41.0	54.4	90.3 (86.1 to 93.7)	70.5 (65.6 to 75.1)	0.804 (0.77 to 0.83)	1.33	N	
	KE	1586	0	1582	53	215	35	1279	5.6	16.9	60.2 (49.2 to 70.5)	85.6 (83.7 to 87.4)	0.729 (0.68 to 0.78)	3.03	N	
Check anaemia (pallor or refer to HB test)	BA	209	0	208	40	6	17	145	27.4	22.1	70.2 (56.6 to 81.6)	96.0 (91.6 to 98.5)	0.831 (0.77 to 0.89)	0.81	Y	*
	CA	633	0	506	215	68	59	164	54.2	56.0	78.5 (73.1 to 83.2)	70.7 (64.4 to 76.5)	0.746 (0.71 to 0.78)	1.03	Y	
	KE	1585	11	1568	125	200	110	1133	15.0	20.7	53.2 (46.6 to 59.7)	85.0 (83 to 86.9)	0.691 (0.66 to 0.72)	1.38	N	
Ask about excessive bleeding	BA	209	0	208	24	11	21	152	21.6	16.8	53.3 (37.9 to 68.3)	93.3 (88.2 to 96.6)			NA	*
	CA	633	0	632	237	150	73	172	49.1	61.2	76.5 (71.3 to 81.1)	53.4 (47.8 to 59)	0.649 (0.61 to 0.69)	1.25	N	
	KE	1586	1	1581	291	226	145	919	27.6	32.7	66.7 (62.1 to 71.2)	80.3 (77.8 to 82.5)	0.735 (0.71 to 0.76)	1.19	Y	
Discuss danger signs for the mother	BA	209	1	208	18	16	31	143	23.6	16.4	36.7 (23.4 to 51.7)	89.9 (84.2 to 94.1)	0.633 (0.56 to 0.71)	0.69	N	
	CA	633	0	632	131	120	80	301	33.4	39.7	62.1 (55.2 to 68.7)	71.5 (66.9 to 75.8)	0.668 (0.63 to 0.71)	1.19	N	
	KE	1586	2	1578	100	184	154	1140	16.1	18.0	39.4 (33.3 to 45.7)	86.1 (84.1 to 87.9)	0.627 (0.60 to 0.66)	1.12	N	
Provider: nurse/midwife	BA	209	0	208	11	1	3	193	6.7	5.7	78.6 (49.2 to 95.3)	99.5 (97.2 to 100.0)			NA	
	KE	1419	0	1414	1100	110	154	50	88.7	85.6	87.7 (85.8 to 89.5)	31.2 (24.2 to 39.0)	0.595 (0.56 to 0.63)	0.96	N	
Provider: doctor/medical officer	BA	209	0	208	141	6	1	60	68.3	70.7	99.3 (96.1 to 100.0)	90.9 (81.3 to 96.6)	0.951 (0.92 to 0.99)	1.04	Y	*
	KE	1419	0	1414	19	108	17	1270	2.5	9.0	52.8 (35.5 to 69.6)	92.2 (90.6 to 93.5)			NA	
Discuss STIs or HIV/AIDS	CA	633	0	632	12	46	7	567	3.0	9.2	63.2 (38.4 to 83.7)	92.5 (90.1 to 94.5)			NA	
	KE	1586	3	1582	72	296	35	1179	6.8	23.3	67.3 (57.5 to 76.0)	79.9 (77.8 to 81.9)	0.736 (0.69 to 0.78)	3.42	N	
Discuss family planning*	BA	209	0	208	19	1	183	5	11.5	9.6	79.2 (57.8 to 92.9)	99.5 (97.0 to 100.0)			NA	*
	CA	633	0	632	210	165	213	44	40.2	59.2	82.7 (77.5 to 87.1)	56.3 (51.2 to 61.4)	0.696 (0.66 to 0.73)	1.48	N	
	KE	1586	0	1585	413	256	164	752	36.4	42.2	71.6 (67.7 to 75.2)	74.6 (71.8 to 77.3)	0.731 (0.71 to 0.75)	1.16	Y	
Newborn care																
Discuss breast feeding/infant feeding	BA	209	0	208	129	11	16	52	69.7	67.3	89.0 (82.7 to 93.6)	82.5 (70.9 to 90.9)	0.858 (0.80 to 0.91)	0.97	Y	*
	CA	605	0	605	428	51	58	68	80.3	79.2	88.1 (84.8 to 90.8)	57.1 (47.7 to 66.2)	0.726 (0.68 to 0.77)	0.99	Y	
	KE	1585	1	1577	752	244	233	348	62.5	63.2	76.3 (73.6 to 79.0)	58.8 (54.7 to 62.8)	0.676 (0.65 to 0.70)	1.01	N	
Examine baby (undressed)	BA	38	0	37	13	2	4	18	45.9	40.5	76.5 (50.1 to 93.2)	90.0 (68.3 to 98.8)			NA	*
	CA	605	0	521	208	151	20	142	43.8	68.9	91.2 (86.8 to 94.6)	48.5 (42.6 to 54.3)	0.698 (0.66 to 0.73)	1.57	N	
	KE	1577	9	1564	345	677	137	405	30.8	65.3	71.6 (67.3 to 75.6)	37.4 (34.5 to 40.4)	0.545 (0.52 to 0.57)	2.12	N	
Weigh the baby	BA	209	0	208	19	2	3	184	10.6	10.1	86.4 (65.1 to 97.1)	98.9 (96.2 to 99.9)			NA	
	CA	605	0	522	352	123	14	33	70.1	91.0	96.2 (93.7 to 97.9)	21.2 (15 to 28.4)	0.587 (0.55 to 0.62)	1.30	N	
	KE	1582	9	1537	987	247	102	201	70.9	80.3	90.6 (88.7 to 92.3)	44.9 (40.2 to 49.6)	0.677 (0.65 to 0.70)	1.13	N	
Give advice/discuss immunisation for the baby	BA	209	7	201	34	6	9	152	21.4	19.9	79.1 (64.0 to 90.0)	96.2 (91.9 to 98.6)	0.876 (0.81 to 0.94)	0.93	Y	*
	CA	605	1	604	407	63	84	50	81.3	77.7	82.7 (79.1 to 86.0)	44.2 (34.9 to 53.9)	0.635 (0.59 to 0.68)	0.96	N	
	KE	1585	3	1576	1013	94	356	113	86.9	70.2	74 (71.6 to 76.3)	54.6 (47.5 to 61.5)	0.643 (0.61 to 0.68)	0.81	N	
Immunise the baby	CA	605	1	604	408	119	23	54	71.4	87.1	94.4 (91.8 to 96.4)	31.2 (24.4 to 38.7)	0.628 (0.59 to 0.66)	1.22	N	*
	KE	1585	8	1564	1207	72	44	241	80.0	81.8	96.5 (95.3 to 97.4)	77 (71.9 to 81.5)	0.867 (0.84 to 0.89)	1.02	Y	
Give information on baby sickness signs	BA	209	7	201	31	3	69	98	49.8	16.9	31.0 (21.1 to 41.0)	97.0 (91.6 to 99.4)	0.640 (0.59 to 0.69)	0.34	N	
	CA	605	1	603	95	151	84	273	29.7	40.8	53.1 (45.5 to 60.6)	64.4 (59.6 to 68.9)	0.587 (0.54 to 0.63)	1.37	N	
	KE	1587	4	1562	181	467	77	837	16.5	41.5	70.2 (64.2 to 75.7)	64.2 (61.5 to 66.8)	0.672 (0.64 to 0.70)	2.51	N	

Estimated prevalence: estimated prevalence that would be obtained from a household survey applying the sensitivity, specificity and prevalence observed in this study.

Benchmark for high validity is AUC >0.7 and IF between 0.75 and 1.25.

See online supplementary tables 1 and 2 for indicator construction and differences across studies.

*In Bangladesh, a question was asked if the mother was informed or advised on child spacing and family planning.

†Number of asterisks refers to number of studies where both criteria were met.

AUC, area under the receiver operating characteristic curve; BA, Bangladesh; CA, Cambodia; FN, false negative; FP, false positive; HB, haemoglobin; IF, inflation factor; KE, Kenya; N, no; NA, not assessed; PNC, postnatal care; STI, sexually transmitted infection; TN, true negative; TP, true positive; Y, yes.

To assess population-level validity for each indicator, we calculated the degree to which an indicator would be overestimated or underestimated in a household survey using the inflation factor (IF). Specifically, the IF is the ratio of the indicator’s estimated population-based survey prevalence to the indicator’s ‘true’ (observed) prevalence. To estimate the population-based survey prevalence (Pr), we applied the indicator’s estimated sensitivity (SE) and specificity (SP) to its true observed prevalence (P), using the following equation: Pr=P×(SE+SP–1)+(1–SP).^28^ We used an IF cut-off between 0.75 and 1.25 as the benchmark for low population-level bias.^28^

Indicators based on a small number of true (observed) positive or true negative cases which resulted in estimated precision for SE or SP of 15 percentage points or more are reported in the data tables, but not discussed in the text. The summary measures AUC and IF are also suppressed for these indicators due to a high degree of uncertainty around the estimate. All analyses were performed using R Studio V.1.1.383 (RStudio, Boston, Massachusetts).

## Results

### Sample description

The characteristics of women who attended an antenatal or postnatal consultation for themselves or their newborn are presented in table 3. Women’s age ranged from 18 to 52 years, with a median age of 24 years (IQR: 21–28) for antenatal clients and a median age of 25 (IQR: 22–30) for postnatal clients. For antenatal clients, approximately half of women in Cambodia and Kenya were attending their first visit for their current pregnancy, while 65% of women were attending their first visit in Bangladesh. Bangladesh and Kenya ANC clients were most likely to be in their third trimester (50% and 49%), whereas 27% of women in Cambodia were in their third trimester. More than one-third of women had one prior pregnancy in all countries (40% in Bangladesh, 36% in Cambodia and 34% in Kenya). Of the women, 15% in Bangladesh, 11% in Cambodia and 5% in Kenya had no school or less than primary school as their highest educational attainment.

**Table 3:** Sample characteristics

	ANC clients						PNC clients					
	Bangladesh		Cambodia		Kenya		Bangladesh		Cambodia		Kenya	
	Total=1036		Total=952		Total=1176		n=208		n=635		n=1619	
	n	%	n	%	n	%	n	%	n	%	n	%
Round												
Baseline	485	46.8	478	49.8	783	66.6	38	18.3	221	34.8	1070	66.1
Follow-up	551	53.2	474	50.2	393	33.4	170	81.7	414	65.2	549	33.9
Age group												
15–19	225	21.7	48	5.1	155	13.5	37	17.8	30	4.7	201	12.8
20–29	668	62.0	641	67.5	736	64.0	139	66.8	415	86.5	976	62.0
30–39	137	34.9	237	25	244	21.2	32	15.4	174	27.5	367	23.3
40+	6	0.6	23	2.4	16	1.4	0	0.0	15	2.4	31	2.0
Education												
None/preprimary	146	14.8	107	11.2	62	5.3	43	21.4	77	12.2	102	6.4
Primary	231	23.3	476	50.0	628	54.0	53	26.4	305	48.2	904	56.4
Secondary	561	56.7	275	28.9	344	29.6	103	50.0	202	31.9	412	25.7
Tertiary	52	5.3	94	9.9	130	11.2	7	3.4	49	7.7	184	11.5
Marital status												
Never married	0	0.0	9	1.0	45	11.6	0	0.0	3	0.5	187	11.7
Ever married	1036	100.0	942	99.0	497	85.1	208	100.0	628	99.5	1415	88.3
Number of times pregnant/given birth*												
1	192	39.6	344	36.4	392	33.7	16	42.1	249	39.6	519	32.8
2	142	29.3	272	28.8	333	28.6	12	31.6	118	29.9	426	26.9
3	78	16.1	149	15.8	202	17.3	5	13.2	100	15.9	288	18.2
4+	73	15.1	179	19.0	238	20.4	5	13.2	92	14.6	351	22.2
Visit number												
First	669	64.6	475	49.9	560	47.7			368	58.0		
Follow-up	366	35.4	477	50.1	613	52.3			267	42.0		
Gestational age of client												
First trimester	96	9.3	179	42.2	157	20.2						
Second trimester	420	40.6	131	30.9	241	31.0						
Third trimester	512	50.1	114	26.9	380	48.8						
Age of baby (weeks)												
<2							40	19.2	361	58.1	174	37.3
2–4							109	52.4	54	8.7	109	23.4
5–8							59	28.4	206	33.2	183	39.3

*Number of times pregnant for ANC and number of times have given birth for PNC. Parity variable not available at follow-up in Bangladesh PNC.

ANC, antenatal care; PNC, postnatal care.

For postnatal clients, 13% of women in Bangladesh, 15% in Cambodia and 22% in Kenya had four or more prior births. The age of the infant was less than 2 weeks for 19% of the sample in Bangladesh, 58% in Cambodia and 37% in Kenya. The proportion of women whose highest education was less than primary school completion was similar to that of ANC clients (21% Bangladesh, 12% Cambodia and 6% Kenya).

### Validation results

#### ANC service coverage indicators

For ANC we assessed 18 indicators in Bangladesh, 5 in Cambodia and 8 in Kenya (table 1). Out of the three countries, the greatest accuracy was observed in Bangladesh: 16 of 18 indicators met both validation criteria. A similar proportion of ANC indicators in Kenya (3 of 8) and Cambodia (3 of 6) met both validation criteria. Across ANC indicators, responses of ‘Don’t Know’ were minimal (<1%) (table 1).

Two indicators were assessed in all three countries: whether the mother was screened for anaemia and whether the fetal heart rate was checked. Both indicators met both validation criteria in two of the three countries. Of the eight indicators assessed in two countries, four indicators—whether the woman’s blood pressure was checked, an abdominal examination was performed, a urine screen was performed and whether a nurse/midwife attended the woman during the consultation—met both validation criteria in both countries where they were tested. Whether the woman’s weight was measured and whether a doctor or medical resident attended the consultation met both criteria in one of two countries. Two indicators of ANC counselling—whether the woman was counselled on the status of her pregnancy and whether the provider gave her a date to return for care—had lower SP (ranging between 21.5% and 54.5% across countries) and did not meet the AUC in either of the two countries where they were tested.

Of 10 indicators unique to Bangladesh, 8 met both validation criteria. Of these indicators, the lowest SE (55.9, 95% CI 49.5 to 62.0) was observed for whether the woman was referred or received ultrasonogram. The lowest SP was observed for whether the woman was informed on possible pregnancy-related complications (69.5, 95% CI 66.4 to 72.6). In contrast, the highest SE (98.0, 95% CI 96.6 to 98.9) and SP (98.0, 95% CI 96.7 to 98.9) were observed for whether a family welfare visitor attended the ANC consultation. This indicator has relevance for both DHS and MICS as the type(s) of provider(s) who attended the consultation is routinely tracked. Notably, whether the woman received a blood test during ANC also had relatively high SE (71.6, 95% CI 66.4 to 76.3) and SP (98.0, 95% CI 96.7 to 98.9). This indicator is currently tracked in both the DHS and MICS core questionnaires.

#### PNC service coverage indicators

For indicators of PNC, we assessed 8 indicators in Bangladesh, 14 in Cambodia and 16 in Kenya. Seven indicators were assessed in all three countries, seven indicators were tested in two countries, and three indicators were tested in one country only. ‘Don’t Know’ responses were minimal for most indicators (≤1%). Two indicators in Bangladesh—whether the provider discussed infant immunisations or gave information on sickness signs for the baby—had slightly higher ‘Don’t Know’ responses of 3% (table 2). Overall, three indicators (two pertaining to maternal PNC and one to newborn care) met both validation criteria in two countries. No indicator (of the seven possible) met both validation criteria in all three countries.

Similar to ANC, indicators tested in Bangladesh had the highest accuracy (6 of 8), followed by 5 of 14 in Cambodia and 3 of 16 in Kenya. The two PNC indicators which did not meet both validation criteria in Bangladesh were whether the provider discussed maternal danger signs for the mother (SE: 36.7, 95% CI 23.4 to 51.7; SP: 89.9, 95% CI 84.2 to 94.1) and whether the provider gave information on baby sickness signs (SE: 31.0, 95% 22.1 to 41.0; SP: 97.0, 95% CI 91.6 to 99.4). Of the five indicators which met both validation criteria in Cambodia, four related to maternal, rather than newborn, care. These were whether the provider checked the mother’s blood pressure, performed a breast examination, conducted an abdominal examination or checked for anaemia. The one validated item pertaining to newborn care was whether the provider discussed breast feeding or feeding for the baby with the mother. Similarly, two of the three indicators in Kenya which met both validation criteria pertained to aspects of the maternal consultation: whether the provider checked for excessive bleeding or discussed family planning with the mother. The one item found to be of acceptable SE and SP in Kenya regarding newborn care was whether the provider immunised the baby.

## Discussion

This study extends prior validation research related to maternal and newborn care by assessing the validity of ANC and PNC service coverage indicators^16^ using data from three additional LMIC settings. Our results make evident that, at discharge, women are able to report with accuracy on multiple aspects of antenatal and postnatal care, particularly in contrast to the very few intrapartum and immediate postnatal (within 1 hour of birth) indicators, that have met validation criteria.^13–17^ However, study findings also demonstrate considerable variability by survey question and setting. Despite this heterogeneity, we identified several broad patterns by type of indicator.

The first is a general pattern of higher reporting accuracy for indicators related to concrete, observable interventions. For example, most ANC indicators which met both accuracy criteria in at least two countries reflected health checks during a physical examination of the mother (blood pressure check, abdominal examination, anaemia screening, urine test and fetal heart rate monitoring). The same trend was observed for PNC indicators. Indicators which reflected physical examination of the mother were more likely than indicators of counselling interventions, provider type or newborn care to meet both validation criteria in half or more of settings tested.

In contrast, ANC and PNC indicators that reflected more abstract concepts, particularly those pertaining to counselling or advice given, performed less reliably. Neither recalling whether the woman was counselled on the status of her pregnancy, given a return date in ANC, nor whether the provider gave information on sexually transmitted infections (STIs) and HIV/AIDS, discussed danger signs for the mother or gave information on the baby’s sickness signs in PNC met both validation criteria in any of the countries tested. For nearly all these indicators, the AUC fell below the benchmark of 0.70, with the exception of being given information on STI and HIV/AIDS. Notably, at the time of data collection in Kenya facilities, there was emphasis on provider-initiated HIV counselling and testing which may have enhanced the quality of counselling provision. These results raise questions around how counselling is conducted and how well women understand and retain the information they are given. Also, what makes counselling memorable to women?

An analysis of DHS service provision assessment data from Haiti, Malawi and Senegal collected between 2012 and 2014 found that the strongest predictor of client ANC knowledge was when client and observer reports agreed that counselling on a given topic had been performed.^29^ This study also found that client and observer agreement that counselling had taken place was generally low and suggested that poor-quality counselling was a factor in client acknowledgement that counselling had occurred. Two exceptions to the relatively low validity of counselling-type indicators documented in this study were counselling related to family planning and whether the provider discussed breast feeding or infant feeding with the mother, which met both validation criteria in two countries, respectively. Notably, these two indicators had higher SE than other counselling-type indicators. It is possible that in these cases counselling was paired with an observable action such as breastfeeding demonstration or being shown or receiving a family planning method.

The more accurate recall of physical aspects of care, as opposed to advice or information given, has mixed support in prior validation studies. A PNC recall study of similar design in Kenya and eSwatini found that five of the same six indicators of maternal physical examination as measured in this study met both validation criteria in at least one of the two countries.^16^ However, the Kenya and eSwatini study also found somewhat better recall of counselling indicators. Two counselling indicators (whether the provider discussed danger signs for the mother or gave information on STIs and HIV) met both validation criteria in either Kenya or eSwatini, whereas neither of these indicators met the benchmark in any setting of the present study. Another study in China which compared women’s reports with facility records found generally lower SP for indicators of the maternal physical examination during ANC and PNC than the present and only prior known PNC validation study.^19^ The China study did not include counselling-type indicators. Notably, women included in the survey had delivered at least one live birth in the country in the 5 years preceding the survey and therefore had a substantially longer recall period.

Another notable trend observed was that indicators related to care for the mother herself were more accurately recalled than indicators of care for the newborn in PNC. Across indicators, SP was on average lower for newborn PNC interventions relative to maternal PNC interventions, demonstrating that women had a tendency to over-report newborn interventions received. The same trend for lower SP was observed in a prior validation study conducted in Kenya and eSwatini for newborn postnatal interventions.^16^ Overall, a similar proportion of newborn indicators met both validation criteria in at least one country in the present study (five of six), as in the McCarthy *et al* study^16^ conducted in Kenya and eSwatini (four of five). Three indicators—whether the provider discussed breast feeding/infant feeding with the mother, weighed the baby or immunised the baby—had acceptable validity in at least one setting in each of the studies.

While broad trends in the types of indicators accurately recalled are apparent, the substantial variation across samples raises questions about what characteristics of the respondent or setting influence recall accuracy. For example, for both ANC and PNC, indicators tended to have the highest SE and SP in Bangladesh, while these were lower in Cambodia and Kenya. These findings lead to questions as to whether differences in respondent characteristics contribute to differences in recall accuracy. For example, are women with a greater number of prior births primed to recall interventions received because they are more knowledgeable about the standard of care? Or does the expectation of type of care that should be received (eg, in a higher-tier facility or in a setting where an intervention is near universally vs rarely practised) matter more? It is also possible that respondent expectations of care based on facility attributes, random variation or differences in data training protocols account for discrepancies by setting. Such questions warrant further investigation and highlight the need for validation research in additional settings.

A strength of this study is the inclusion of a large number and range of type of facilities in each setting. Greater variation in facility practices addresses sample size limitations due to lack of variation in facility practices in prior validation research.^13 14 16^ Examination of validation results in three different countries also gives insight into how individual validity may vary by setting and which indicators tended to perform most consistently. However, this study also has several limitations. While direct observation by a third-party observer is considered to be gold standard, it may also be imperfect. Differences in observer training protocols, facility practices or how apparent it was that a given intervention was implemented, among other factors, may contribute to differences in observer ratings across settings. For example, observation of counselling interventions may have been more subjective regarding whether counselling took place. This may have contributed to lower SE and SP for counselling-type indicators. Finally, summary accuracy criteria should be interpreted with care. Global measures of test accuracy fail to distinguish between false negative and false positive test errors. We also caution against generalising the population-based results of this study to other settings, depending on the prevalence of the intervention. We have previously demonstrated how indicator properties established in this study can be extended to contexts with varying levels of intervention coverage.^13^

An important consideration of the relevance of study findings for national and global monitoring efforts is that this study assessed women’s immediate recall accuracy (at facility discharge). Results may not be directly generalisable to the DHS and MICS, which typically ask women to recall events related to a birth in the 2–5 years prior. While immediate recall may represent best-case scenario in terms of accuracy, findings inform the degree to which women perceived specific interventions took place. Prior evidence suggests that, unless interventions are recalled with high accuracy at facility discharge, recall generally declines with time. For example, validation analysis of facility-based interventions received in the intrapartum and immediate postnatal periods in Kenya showed that the few select interventions which were recalled with high accuracy at facility discharge maintained acceptable accuracy at 13–15 months’ follow-up.^15^ However, for most indicators, recall accuracy was poor and either remained the same or declined with time. Another study which assessed maternal recall of infant birth weight among women in Nepal at recall periods ranging from 1 to 24 months postdelivery found that recall was generally poor and relatively uninfluenced by length of follow-up.^30^ While additional research evaluating different lengths of recall time is warranted, it is possible that high immediate recall is necessary in order to ‘code’ certain events into memory for later reporting.

In the context of calls for enhanced measurement of the components that lead to effective coverage, study findings such as these suggest that careful consideration of the type of information women are asked to recall is needed. While household survey programmes such as the DHS and MICS are frequently relied on as data sources for measuring intervention coverage, findings should be triangulated with other data sources such as routine data from health information systems. As new indicators are proposed, they should be subject to validity tests in a range of settings and recall periods. Variations in question wording and sequence should be systematically tested to investigate whether validity can be improved.

## Conclusion

This study extends the scant evidence base on aspects of antenatal and postnatal interventions that women can accurately recall and report on in household surveys. In contrast to prior validation studies of intrapartum and immediate PNC (within 1 hour of birth), we find women are able to recall with accuracy some aspects of antenatal and routine PNC. While we note some trends in reporting accuracy, such as generally more accurate recall for indicators related to observable (eg, maternal physical examination) rather than counselling (eg, discussion of STIs or HIV) interventions, considerable variability in results by survey question and setting is also evident.
