# Supplementary material for: Validating women’s reports of antenatal and postnatal care received in Bangladesh, Cambodia and Kenya
Source: BMJ Glob Health. 2020 Apr 19;5(4):e002133. doi: 10.1136/bmjgh-2019-002133 (PMC7245420; doi:10.1136/bmjgh-2019-002133)
Supplement: Supplementary data [file bmjgh-2019-002133supp001.pdf]

Supplemental Table 1. ANC Indicator construction

Indicator		Bangladesh	Cambodia	Kenya	Tracked in DHS or MICS	Global Strategy/ Initiative
Measure weight	Client	During your visit today, did the staff…take your weight?	During your visit today, did the staff…take your weight?	During your visit today, did the staff…take your weight?	DHS Suppl. Module on Maternal Health Care	
	Observer	Take client's weight	Take client's weight	Take client's weight		
Blood pressure check	Client	Check your blood pressure	Check your blood pressure	Check your blood pressure	DHS Core Questionnaire; MICS Questionnaire	EPMM (additional priority indicator for development); Countdown to 2030 B9
	Observer	Take client's blood pressure	Take client's blood pressure	Take client's blood pressure		
Examine abdomen	Client	Examine lower abdomen for fetal presentation	Perform abdominal examination	Perform abdominal examination		
	Observer	Palpate abdomen for fetal presentation	Palpate the client's abdomen for fundal height OR Palpate abdomen for fetal presentation OR Palpate abdomen for engagement of presenting parts	Palpate the client's abdomen for fundal height OR Palpate abdomen for fetal presentation OR Palpate abdomen for engagement of presenting parts		
Check anemia (pallor or refer for HB test)	Client	[Baseline] Check for anemia [Follow-up] Check conjunctiva (eyelids) OR Blood test for hemoglobin	Check for anemia	Check for anemia		
	Observer	Check conjunctiva or refer/test blood	Observer/test for anemia OR Check the woman's conjunctiva (eyelids)	Observe/test for anemia OR Check the woman's conjunctiva (eyelids)		
Check fetal heart rate	Client	Check fetal pulse rate	Listen to the baby's heartbeat	Listen to the baby's heartbeat	DHS Suppl. Module on Maternal Health Care	
	Observer	Listen to fetal heart rate	Listen to fetal heart rate	Listen to fetal heart rate		
Urine screen	Client	[Baseline]: Had lab test (urine); [Follow-up] Lab test (urine) for protein OR Lab test (urine) for glucose OR Lab test (urine) for albumin OR Lab test (urine) for	Check urine for protein and sugar	Check urine for protein and sugar	DHS Core Questionnaire; MICS Questionnaire	EPMM (additional priority indicator for development); Countdown to 2030 B9

		others				
	Observer	Urine test or refer client for urine bacterium	Urine test or refer client for urine bacterium	Urine test or refer client for urine bacterium		
Inform status of pregnancy	Client	NA	Talk with you about the progress of your pregnancy	Talk with you about the progress of your pregnancy	DHS Suppl. Module on Maternal Health Care (Asked whether discussed with a health provider when the baby is due or will arrive)	
	Observer	Inform the client about the progress of the pregnancy	Inform the client about the progress of the pregnancy	Inform the client about the progress of the pregnancy		
Give return date	Client	NA	Did the provider tell you to come back for another visit?	Did the provider tell you to come back for another visit?		
	Observer	NA	Give the client the return date?	Give the client the return date?		
Provider - nurse/midwife	Client	Nurse/midwife/paramedics	NA: 'Health provider'	Nurse/midwife	Type of provider in DHS Suppl. Module on Maternal Health Care	Countdown to 2030 B7
	Observer	Nurse/senior nurse	Designation of observed provider: Primary/secondary nurse OR primary/secondary nursemidwife	Registered/enrolled nurse/midwife or BSC nurse		
Provider - doctor	Client	Gynae consultant/medical officer/ MBBS Do	NA: 'Health provider'	Doctor (general)	Type of provider in DHS Suppl. Module on Maternal Health Care	
	Observer	Gynae consultant/mo	Doctor	Medical officer/doctor		
Take pulse	Client	Check pulse	NA	NA		
	Observer	Check pulse				
Check temperature	Client	[Baseline] Body temperature/Fever [Follow-up] Check temperature	NA	NA		
	Observer	Measure body temperature				
Checked edema	Client	Check for edema	NA	NA		
	Observer	Check for edema				
Vaginal	Client	Internal examinations	NA	NA	DHS Suppl.	

examination	Observer	Perform internal examination for vaginal discharge			Module on Maternal Health Care* (Asked whether had vaginal bleeding)	
Blood test	Client	During this antenatal checkup did you have any of the following laboratory exams? - Blood test	NA	NA	DHS Core Questionnaire; MICS Questionnaire	EPMM (additional priority indicator for development); Countdown to 2030 B9
	Observer	Perform/refer for blood test				
Ultrasonogram	Client	Ultrasound performed	NA	NA		
	Observer	Perform/refer for Ultrasonogram				
Advise on diet/nutrition	Client	Give advice on diet and nutrition	NA	NA	DHS Core Questionnaire* (During this pregnancy, were you given or did you buy any iron tablets or iron syrup?)	Countdown to 2030 B23
	Observer	Advice on quantity and quality of food to eat during pregnancy				
Advise on TT injection	Client	Inquire about TT injection history	NA	NA	DHS/MICS Core Questionnaire* (During this pregnancy, were you given an injection in the arm to prevent the baby from getting tetanus, that is, convulsions after birth?)	
	Observer	Advise on TT injection				
Discuss care for breasts/ breastfeeding	Client	Discuss care for breasts/ breastfeeding	[Follow-up only] Discuss with you how to care for the breasts	NA		
	Observer	Advise on usefulness of breastfeeding/taking care of breasts OR Advise client on care of breasts	[Follow-up only] Advise client on care of breasts			

Inform on possible pregnancy related complications	Client	Inform you about possible pregnancy related complications	NA	NA	DHS Suppl. Module on Maternal Health Care* (Told by a health worker about danger signs that might indicate problems with the pregnancy?)
	Observer	Did the provider inform or advise the following signs as risk factors during pregnancy period for which the woman should return immediately to the health facility?			
Family welfare visitor attended ANC	Client	Family welfare visitor	NA	NA	Type of provider in DHS Suppl. Module on Maternal Health Care
	Observer	Senior family welfare visitor/ family welfare visitor			
Medical Asst. attended ANC	Client	Medical assistant	NA	NA	Type of provider in DHS Suppl. Module on Maternal Health Care
	Observer	Sacmo/medical assistant			

Supplemental Table 2: PNC indicator construction.

		Bangladesh	Cambodia	Kenya	Tracked in DHS or MICS	Global Strategy
Blood pressure check	Client	Measure your blood pressure	Measure your blood pressure	Measure your blood pressure	DHS Suppl. Module on Maternal Health Care (In first two days of birth, did any health provider measure your blood pressure?)	Overlap with C12 and C13 (Content of PNC for mothers and newborns)
	Observer	Take client's blood pressure	Take client's blood pressure	Take client's blood pressure		
Breast exam	Client	Examine your breasts	Examine your breasts	Examine your breasts		
	Observer	Examined breasts and nipples	Examined breasts and nipples	Examined breasts and nipples		
Examine abdomen	Client	Examine height of uterus	Examine your abdomen	Examine your abdomen		
	Observer	Lower abdominal examination for uterine involution	Palpate the client's abdomen for uterine involution	Palpate the client's abdomen for uterine involution		
Examine vagina	Client	Did you have vaginal bleeding/perineal/episiotomy examination in PNC	Examine the vagina	Examine the vagina		
	Observer	Checked/asked about extent of vaginal bleeding/perineal tear/examined episiotomy/ checked vaginal discharge	Pelvic examination /Checked perineum	Pelvic examination /Checked perineum		
Check anemia (pallor or refer for HB test)	Client	Did you have an anemia examination?	Check you for anemia	Check you for anemia		
	Observer	Checked woman's conjunctiva for anemia	Check for pallor (anemia) /Refer for anemia test (HB)	Check for pallor (anemia) /Refer for anemia test (HB)		
Ask about excessive bleeding	Client	Did you have a vaginal bleeding examination?	Ask if you had any abnormal bleeding	Ask if you had any abnormal bleeding	DHS Suppl. Module on Maternal Health Care (In first two days of birth, did any health provider examine for or ask you about vaginal bleeding?)	
	Observer	Checked/asked about extent of peri-vaginal bleeding [Or] Checked if bleeding since birth	Checked/asked about extent of peri-vaginal bleeding	Checked/asked about extent of peri-vaginal bleeding		
Discuss danger signs for mother	Client	Inform on possible PNC related problems/ complications	Discuss with you signs of complications after birth	Discuss with you signs of complications after birth		

	Observer	Discussed any of following: foul smelling vaginal discharge, fever with or without chills, excessive vaginal bleeding, broken stitches (perineal), cracked nipples, painful engorged breasts	Discussed any of following: foul smelling vaginal discharge, fever with or without chills, excessive vaginal bleeding, broken stitches (perineal), cracked nipples, painful engorged breasts	Discussed any of following: foul smelling vaginal discharge, fever with or without chills, excessive vaginal bleeding, broken stitches (perineal), cracked nipples, painful engorged breasts	
Provider-nurse/midwife	Client	Who attended you? Nurse/midwife	Who attended you? NA - Health provider.	Who attended you? Nurse/midwife	DHS Core Questionnaire (type of provider)
	Observer	Designation of observed provider. Nurse/midwife	Primary midwife, Secondary midwife	Registered nurse/midwife; Enrolled nurse/midwife, BSC nurse	
Provider-doctor/medical officer	Client	Med officer/ Gyn / MBBS/ MBBS attended PNC	Who attended you? NA - Health provider.	Doctor (general)	DHS Core Questionnaire (type of provider)
	Observer	Med officer/ Gyn / MBBS attended PNC	Doctor	Doctor /medical officer	
Discussed with you family planning	Client	Did the service provider inform or advice on… child spacing or use of family planning after delivery? [Or] [Or] Various family planning methods?	Discuss with you family planning	Discuss with you family planning	DHS Suppl. Module on Maternal Health Care (In first two days of birth, did any health provider counsel you about methods to prevent pregnancy?)
	Observer	Discuss healthy timing and birth spacing or use family planning after delivery [Or] Discuss health benefits for mother and baby when birth spacing [Or] Discuss various family planning methods	Discuss healthy timing and spacing of pregnancies /family planning [Or] Discuss or advise on the various family planning methods?	Discuss healthy timing and spacing of pregnancies /family planning [Or] Discuss or advise on the various family planning methods?	
Discussed STIs or HIV/AIDS	Client	NA	Give information or advice on sexually transmitted infections or the AIDS virus	Give information or advice on sexually transmitted infections or the AIDS virus	
	Observer	NA	Discuss HIV/AIDs with client? [Or] Discuss STIs with the client	Discuss HIV/AIDs with client? [Or] Discuss STI/HIV risk factors with the client?	

Discuss breastfeeding/feeding for baby	Client	Counseled on breastfeeding for baby? [Or] Discuss with you about breastfeeding?	Discuss breastfeeding/feeding for the baby	Discuss breastfeeding/feeding for the baby	DHS Core Questionnaire; MICS Questionnaire (During first two days after birth, did any health provider counsel you on breastfeeding?)	Countdown to 2030 (Aspirational) C
	Observer	Yes to any of following: Discussed infant feeding, Encouraged mother to discuss how she was managing with breastfeeding, Re-emphasized exclusive feeding, Emphasized NO to mixed feeding	Yes to any of following: Discussed infant feeding, Encouraged mother to discuss how she was managing with breastfeeding, Re-emphasized exclusive feeding, Emphasized NO to mixed feeding	Yes to any of following: Discussed infant feeding, Encouraged mother to discuss how she was managing with breastfeeding, Re-emphasized exclusive feeding, Emphasized NO to mixed feeding		
Examine baby (undressed)	Client	Physical examination of baby	Examine the baby (physical check, unclothed)	Examine the baby (physical check, unclothed)		
	Observer	Examine the baby (undressed)	Examine the baby (undressed)	Examine the baby (undressed)		
Weigh the baby	Client	Baby's weight measured	Baby's weight measured	Baby's weight measured		
	Observer	Did the provider weigh the baby	Did the provider weigh the baby	Did the provider weigh the baby		
Give advice/discuss immunizations	Client	Advice to immunize the baby	Give you advice about immunizations for the baby	Give you advice about immunizations for the baby		
	Observer	Did the provider discuss immunizations for the baby	Discuss immunizations for the baby?	Discuss immunizations for the baby?		
Immunize baby	Client	NA	Immunize the baby	Immunize the baby		
	Observer	NA	Give the baby immunizations?	Give the baby immunizations?		
Gave information on baby's sickness signs	Client	Counseled on sickness signs for baby	Give you information on the baby's sickness signs	Give you information on the baby's sickness signs	DHS Core Questionnaire (During first two days after birth, did provider counsel you on danger signs for newborns?); MICS Questionnaire (During the first two days after birth, did any health care	
	Observer	Discussed any of following: difficulty in breathing; poor feeding; jaundice (yellow skin/eyes); bleeding from cord; redness, swelling and/or pus around cord; baby feels hot or cold; abnormal crying; abdominal	Discuss infant danger signs (any one of following): feeding difficulties- not sucking or sucking poorly, breathing difficulties, body feels hot or too cold, jaundice - yellow	Discuss infant danger signs (any one of following): feeding difficulties- not sucking or sucking poorly, breathing difficulties, body feels hot or too cold, jaundice - yellow		

	distension/vomiting; septic spots/boils on body,; lethargy; convulsions	skin or eyes.	skin or eyes.	provider give you information about the symptoms that require you to take your sick child to a health facility for care?)
